# Selective ablation of mast cells or basophils in mice reduces peanut-induced anaphylaxis

**DOI:** 10.1186/2045-7022-3-S3-P82

**Published:** 2013-07-25

**Authors:** LL Reber, T Marichal, K Mukai, A Roers, K Hartmann, H Karasuyama, KC Nadeau, M Tsai, SJ Galli

**Affiliations:** 1 Department of Pathology, Stanford University, Stanford, CA, USA; 2 Department of Dermatology, University Hospital Cologne, Cologne, Germany; 3 Department of Immune Regulation, Tokyo Medical and Dental University Graduate School, Tokyo, Japan; 4 Department of Pediatrics, Stanford University, Stanford, CA, USA

## Background

Studies using c-*kit* mutant mast cell (MC)-deficient mice and antibody-mediated depletion of basophils have suggested that both MCs and basophils can contribute to peanut-induced anaphylaxis (PIA) in mice. However, interpretation of data obtained using such approaches to analyze the contributions of individual effector cells to active anaphylaxis is complicated since mice with mutations affecting c-*kit* structure or expression have several phenotypic abnormalities in addition to their MC deficiency and basophil-depleting antibodies can also react with MCs.

## Methods

Various mutant mice and the corresponding wild-type mice were orally sensitized with peanut extract and cholera toxin weekly for 4 weeks and challenged intraperitoneally with peanut extract 2 weeks after the last sensitization.

## Results

Upon peanut challenge, peanut-sensitized MC-deficient *Kit^W-sh/W-sh^* mice developed reduced immediate hypothermia compared to identically treated wild-type mice, as well as a late phase hypothermia that was abrogated by antibody-mediated depletion of neutrophils. Diphtheria toxin-mediated selective depletion of MCs or basophils in *Mcpt5-Cre; iDTR* or *Mcpt8^DTR^* mice, respectively, and treatment of wild-type mice with the basophil-depleting antibody Ba103, that recognizes CD200R3 (expressed on both basophils and MCs), significantly reduced but did not fully eliminate peanut-induced hypothermia in peanut-sensitized mice. Peanut sensitized MC- and basophil-deficient *Cpa3-Cre; Mcl-1^fl/fl^* mice, which lack mutations in c-*kit*, developed reduced, but still significant, hypothermia responses to peanut challenge.

## Conclusion

Inducible and selective ablation of MCs or basophils in non-c-*kit* mutant mice can significantly reduce PIA, but partial responses to peanut challenge can still be observed in the virtual absence of either cell type, or in mice (*Cpa3-Cre; Mcl-1^fl/fl^* mice) that virtually lack MCs and have a marked reduction in basophils. The increased levels of neutrophils in *Kit^W-sh/W-sh^* mice may contribute to the hypothermia induced in these mice in this PIA model. Our data suggest that the hypothermia observed in this PIA model in various strains of mice can reflect contributions from MCs, basophils, and neutrophils.

## Disclosure of interest

None declared.

